# Blood metabolic and physiological profiles of Bama miniature pigs at different growth stages

**DOI:** 10.1186/s40813-022-00278-7

**Published:** 2022-08-08

**Authors:** Jiayuan Mo, Yujie Lu, Tianqi Xing, Di Xu, Kun Zhang, Shuai Zhang, Yubin Wang, Gang Yan, Ganqiu Lan, Jing Liang

**Affiliations:** College of Animal Science and Technology, Guangxi University, Nanning, 530004 People’s Republic of China

**Keywords:** Blood profiles, Non-targeted metabolomics, Differential metabolites, Blood physiological indices, Bama miniature pig

## Abstract

**Background:**

Bama miniature pigs aged between six (6 M) and twelve months (12 M) are usually used in human medical research as laboratory pigs. However, the difference in serum metabolic profiles from 6 to 12 M-old pigs remains unclear. This study aimed to identify the metabolic and physiological profiles present in the blood to further explain changes in Bama miniature pig growth. We collected blood samples from 6 M-, eight-month- (8 M-), ten-month- (10 M-), and 12 M-old healthy Guangxi Bama miniature pigs. A total of 20 blood physiological indices (BPIs) were measured: seven for white blood cells, eight for red blood cells, and five for platelet indices. Liquid chromatography and mass spectrometry-based non-targeted metabolomic approaches were used to analyze the difference in metabolites. The associations between the differences were calculated using Spearman correlations with Benjamini–Hochberg adjustment. The 100 most abundant differential metabolites were selected for analysis of their metabolic profiles.

**Results:**

There were no significant differences in BPIs at different ages, but the mid cell ratio and red blood cell number increased with age. Seven BPIs in Bama miniature pigs were closer to human BPIs than to mouse BPIs. A total of 14 and 25 significant differential metabolites were identified in 6 M vs. 12 M and 8 M vs. 12 M, respectively. In total, 9 and 18 amino acids and their derivatives showed significantly lower concentrations in 6 M- and 8 M-old pigs than in 12 M-old pigs. They were identified as the core significantly different metabolites between the age groups 6 M vs. 12 M and 8 M vs. 12 M. Half of the enriched pathways were the amino acids metabolism pathways. The concentration of six amino acids (dl-tryptophan, phenylacetylglycine, muramic acid, *N-*acetylornithine, l(−)-pipecolinic acid, and creatine) and their derivatives increased with age. A total of 61 of the top 100 most abundant metabolites were annotated. The metabolic profiles contained 14 amino acids and derivatives, six bile acids and derivatives, 19 fatty acids and derivatives, and 22 others. The concentrations of fatty acids and derivatives were found to be inversely proportional to those of amino acids and derivatives.

**Conclusion:**

These findings suggest high levels of MID cell ratio, red blood count, and amino acids in 12 M-old pigs as indicators for improved body function over time in Bama miniature pigs, similar to those in human development. This makes the pig a more suitable medical model organism than the mouse. The results of this study are limited to the characteristics of blood metabolism in the inbred Bama miniature pigs, and the effects of impacting factors such as breed, age, sex, health status and nutritional level should be considered when studying other pig populations.

**Supplementary Information:**

The online version contains supplementary material available at 10.1186/s40813-022-00278-7.

## Background

The pig is not only an important domestic animal but also one of the principal model organism for human biomedical research [1]. More precisely, their use includes studies on radiofrequency ablation [2], infected scalds [3], *Bungarus multicinctus* bites [4], and drug development [5]. Bama miniature pigs are easy to handle because of their small size and their anatomical and physiological traits are similar to those in humans. In previous studies, the age of the Bama miniature pigs ranged from six months (6 M) to twelve months (12 M) [6–8]. However, the differences between the physiological characteristics of 6 M- to 12 M-old Bama miniature pigs remain unclear.

Calculating the blood physiological index (BPI) is a good method to analyze the health status of the body by measuring the number and distribution of blood cells [9], including white blood cells (WBC), red blood cells (RBC), and platelet indices [10]. The BPI is not only related to health levels but also related to the age of an animal [11]. Wang et al. reported that the WBC, RBC, and lymphocyte numbers were enhanced in weanling pigs after a 42-d feeding [12]. Okoro et al. found that the RBC number in post-weaning pigs was higher than in pre-weaning pigs [13]. Pliszczak-Król et al. found that the platelet number was at its maximum in four-week-old piglets and reached its minimum at week 18 [14]. In general, a high platelet number is a sign of inflammation [15, 16] and disease [17]. Thus, normal BPIs play a key role in pig health and growth. As the carrier of nutrients, waste, oxygen, and carbon dioxide, the blood contains not only various blood cells but also several molecules in serum, such as amino acids and fatty acids. Teodoro-Morrison et al. reported that the concentrations of amino acids arginine, isoleucine, and leucine were higher in adolescence than in childhood [18]. Wu et al. reported that the amino acid metabolism changes in association to age in mice [19]. This has also found to be true in humans [20, 21]. These findings suggest that amino acid abundance in mammals may increase with age. Thus, the serum metabolites pofiles between 6 M- and 12 M-old Bama miniature pigs may difference.

Metabolomics can be used to qualitatively and quantitatively analyze all small molecule metabolites (< 1 kDa) in specific biological samples (such as serum and urine). Metabolomics has widely been used since the advent of next-generation sequencing technology in pig research. It has been used to identify differences in boar sperm freezability [22], to identify the complex molecular regulatory network of meat quality in Enshi black pigs [23] and the growth mechanism of protein-restricted pigs [24].Thus, the metabolomics is suitable for our study to identify the serum difference between 6 to 12 M Bama miniature pigs.

To determine the general health status, identify metabolic changes, and further explain the changes in growth in 6 M- to 12 M-old Bama miniature pigs, we analyzed the BPIs by measuring their WBC, RBC, and platelet number. In particular, we used non-targeted ultra-high-performance liquid chromatography–high-resolution mass spectrometry (UPLC-MS) to identify the metabolite profiles in the serum of 6 M-, 8 M-, 10 M-, and 12 M-old Bama miniature pigs. This study furthers our understanding of the metabolic changes during pig growth and development, and constitutes a reference for similar studies on humans.

## Materials and methods

### Animals and sample collection

The Bama Xiang miniature pig is a long-term inbred model pig at the Guangxi Bama miniature pig breeding center (Guangxi University, Nanning, Guangxi, China). A total of 68 healthy Guangxi Bama miniature pigs of age six months (6 M: n = 17), eight months (8 M: n = 15), ten months (10 M: n = 18), and twelve months (12 M: n = 18) were randomly selected from the breeding center. All pigs were raised under food and drink freely with the same diet. A total of 10 mL of blood was collected from the anterior vena cava. Collection, handling and transport of the blood samples was done in accordance with the guidelines established by the American Society for Veterinary Clinical Pathology (ASVCP) [25, 26].

### Blood physiological indices measurement

The blood was used to measure the BPIs using the automatic blood cell analyzer PE-6800VET (Procan Electronics Co, Ltd, Shenzhen, China), including WBC number, lymphocyte ratio, MID cell ratio, neutrophil ratio, lymphocyte number, MID cell number, neutrophil number, RBC number, hemoglobin concentration, hematocrit, mean corpuscular volume, mean corpuscular hemoglobin, mean corpuscular hemoglobin concentration, platelet number, mean platelet volume, platelet distribution width, platelet crit, and platelet large cell ratio. All data were statistically analyzed using IBM SPSS statistics for Windows, version 19.0 (IBM Corp., Armonk, NY, USA) and the results were depicted as mean ± SD. Unless otherwise stated, differences were considered statistically significant at *P* < 0.05.

### Serum sample preparation

Six Guangxi Bama miniature pigs (three male and three female) were randomly selected to separated serum by centrifugation (3000 rpm, 20 min, 4 °C). A total of 100 µL serum and 300 µL acetonitrile was added to 1.5 mL Eppendorf tubes, vortexed for 15 s, and then centrifuged (13,000 rpm, 10 min, 4 °C) to precipitate protein. The supernatant fractions were collected and evaporated using a vacuum concentrator. The dry residues were dissolved in a 100 µL solution of acetonitrile and water (in the ratio of 3:1) and centrifuged again (13,000 rpm, 10 min, 4 °C). Finally, the liquid supernatant was transferred to sampler vials for analysis using a UPLC-MS system housed at Guangxi University [27].

### UPLC-MS instrument and parameters

The Dionex liquid chromatography instrument (UltiMate3000, Thermo Fisher Scientific, USA), autosampler (WPS-3000SL, Thermo Fisher Scientific, USA), liquid phase pump (HPG-3400 SD, Thermo Fisher Scientific, USA), column temperature box (TCC-3000 SD, Thermo Fisher Scientific, USA), and Hypersil GOLD C18 column (50 mm × 2.1 mm, 1.7 µm) were used in this study. The ion source was a quadrupole electrostatic field orbit trap high-resolution mass spectrometer Q-Exactive (Thermo Fisher Scientific, USA) heated electrospray (Thermo Fisher Scientific, USA). The scanning mode was full mass spectrum (MS) and full MS/dd-MS2 (Additional file 1: Table S1). The changes of solvents in the gradient elution of UPLC-MS/MS analysis were shown in Additional file 2: Table S2.

### Data analysis

The peak extraction, peak alignment, retention time correction, peak area extraction, accurate mass matching (< 25 ppm), and secondary spectra matching were performed using Compound Discoverer software, version 3.1 (Thermo Scientific). The unsupervised principal component analysis (PCA) and supervised orthogonal partial least squares discriminant analysis (OPLS-DA) were executed using SIMCA-P software, version 14.1 (Umetrics AB, Umea, Sweden). The metabolites which met variable importance in projection (VIP) scores (VIP > 1), fold changes (FC > 2 or FC < 0.5), and *P*-value (*P* < 0.05) were defined as the significantly differential metabolites. The potential disordered metabolic pathways were screened using the Encyclopedia of Genes and Genomes in the Kyoto Protocol (KEGG, http://www.genome.jp/kegg/), the human metabolome database (HMDB, https://hmdb.ca/metabolites/), and MetaboAnalyst 5.0 (https://www.metaboanalyst.ca/). The relationship of the relative concentrations of the before identified significant differential metabolites was calculated using Spearman correlations in R, and the false discovery rate was applied. The adjusted *P* values of < 0.05 and absolute value of > 0.7 were defined as the thresholds for identified relationship pairs to be significant. The co-occurrence network was constructed using Cytoscape software, version 3.8.2. Metabolites that had earlier been identified as being significantly different, and that also had a significant relationship with over 70% of the differential metabolites, were defined as the core significant differential metabolites. The trend analysis was performed using STEM software. The 100 most abundant metabolites from all metabolites were selected and analyzed, to obtain the metabolic profile, which was annotated using HMBD. They were grouped into four types: (1) amino acid and derivatives, (2) bile acid and derivatives, (3) fatty acid and derivatives, and (4) others.

## Results

### Blood physiological index analysis

There was no significant difference in BPIs such as WBC number, lymphocyte number, platelet number, and some other indices (*P* > 0.05) at different ages. However, the lymphocyte ratio at 10 M was significantly higher than that at 6 M and 12 M, and the ratio at 8 M was significantly higher than that at 6 M (*P* < 0.05). The MID cell ratio at 12 M was significantly higher than that at 6 M (*P* < 0.01) and that at 10 M was significantly higher than at 6 M (*P* < 0.05). The neutrophil ratio at 10 M was significantly lower than that at 6 M (*P* < 0.01), and that at 8 M was significantly lower than at 6 M (*P* < 0.05). The MID cell number at 12 M was higher than that at 6 M (*P* < 0.05). The RBC number at 12 M was significantly lower than that at 6 M and 8 M (*P* < 0.01), and at 12 M was much lower than at 10 M (*P* < 0.05). The hemoglobin concentration at 12 M was substantially lower than at 8 M (*P* < 0.01), and that at 12 M was lower than that at 6 M and 10 M (*P* < 0.05). The hematocrit at 12 M was substantially lower than at 8 M (*P* < 0.01), and that at 12 M was lower than that at 6 M (*P* < 0.05) (Table 1). The BPIs of adult humans [28], along with the reference ranges of mouse BPIs and pig BPIs from the animal blood cell analyzer are shown in Table 1.

**Table 1:** The blood physiological indexes in different age

Team	6 month old	8 month old	10 month old	12 month old	Adult human reference	Mouse reference	Pig reference
Number	17	15	18	18			
White blood cell number/(10^9^ L^−1^)	22.46 ± 1.47	20.49 ± 1.72	19.21 ± 1.51	23.77 ± 2.11	3.6–8.9	0.8–6.8	11.0–22.0
Lymphocyte ratio/%	28.61 ± 1.54^c^	32.53 ± 1.60^ab^	33.37 ± 0.55^a^	29.57 ± 0.93^bc^	–	55.8–90.6	39.0–62.0
Mid cell ratio/%	16.72 ± 0.81^Bb^	18.13 ± 0.99^ABab^	19.61 ± 0.74^ABa^	20.55 ± 0.79^Aa^	–	1.8–6.0	2.0–10.0
Neutrophil ratio/%	54.67 ± 2.10^Aa^	49.33 ± 1.80^ABb^	47.03 ± 0.84^Bb^	50.11 ± 1.12^ABab^	36.3–72.9	8.6–38.9	28.5–64.0
Lymphocyte number/(10^9^ L^−1^)	6.48 ± 0.57	6.52 ± 0.50	6.42 ± 0.51	6.86 ± 0.57	18.8–48.0	0.7–5.7	5.5–11.1
Mid cell number/(10^9^ L^−1^)	3.76 ± 0.29^ab^	3.65 ± 0.31^b^	3.71 ± 0.29^ab^	4.68 ± 0.40^a^	–	0.1–0.3	0.6–1.3
Neutrophil number/(10^9^ L^−1^)	12.22 ± 1.00	10.31 ± 1.10	9.08 ± 0.77	12.23 ± 1.24	1.5–6.6	0.1–1.8	0.5–10.0
Red blood cell number/(10^12^ L^−1^)	6.70 ± 0.36^Bb^	6.56 ± 0.21^Bb^	6.93 ± 0.24^ABb^	7.79 ± 0.19^Aa^	3.8–5.4	6.36–9.42	5.00–9.50
Hemoglobin concentration (g L^−1^)	131.71 ± 8.00^ABb^	124.40 ± 5.04^Bb^	129.78 ± 6.07^ABb^	151.39 ± 4.95^Aa^	115–167	11–14	99–165
Hematocrit/%	40.58 ± 2.08^ABb^	37.17 ± 2.75^Bb^	41.28 ± 1.57^ABab^	46.61 ± 1.26^Aa^	34.6–48.8	34.6–44.6	32.0–50.0
Mean corpuscular volume/fL	60.85 ± 0.65	60.30 ± 0.43	59.48 ± 0.41	59.84 ± 0.38	82.9–96.9	48.2–58.3	51.0–68.0
Mean corpuscular hemoglobin/pg	19.47 ± 0.20	18.84 ± 0.20	18.61 ± 0.48	19.33 ± 0.21	–	15.8–19.0	17.0–22.0
Mean corpuscular hemoglobin concentration (g L^−1^)	321.24 ± 5.05	313.47 ± 2.99	313.72 ± 7.72	323.50 ± 2.41	320–361	30–35	300–380
Red blood cell distribution width standard deviation/%	29.82 ± 0.68	28.98 ± 0.35	29.01 ± 0.37	29.61 ± 0.28	–	0.1–99.9	0.1–99.9
Red blood cell distribution width coefficient of variation/%	16.65 ± 25.08	16.28 ± 0.17	16.57 ± 0.19	16.76 ± 0.13	–	13.0–17.0	14.0–19.0
Platelet number (10^9/L)	708.94 ± 53.29	581.20 ± 75.12	623.33 ± 68.32	709.00 ± 52.85	163–336.8	450–1590	200–700
Mean platelet volume/fL	9.55 ± 0.38	8.77 ± 0.27	9.16 ± 0.26	9.49 ± 0.28	7.1–9.5	3.8–6.0	6.0–12.0
Platelet distribution width/%	9.42 ± 0.43	8.69 ± 0.27	9.01 ± 0.18	8.92 ± 0.21	–	0.1–30.0	0.1–30.0
Platelet crit/%	0.69 ± 0.07	0.52 ± 0.07	0.58 ± 0.07	0.62 ± 0.06	–	0.01–9.99	0.01–9.99
Platelet large cell ratio/%	16.89 ± 1.14	16.96 ± 1.06	19.70 ± 1.75	20.36 ± 1.64	–	0.1–99.9	0.1–99.9

The different capital letters in the same rows showed statistically differences at *P* < 0.01; the different lowercase letters showed statistically differences at *P* < 0.05; THE same letter showed that the difference is not statistically significant (*P* > 0.05)

### Scanning of metabolites

In this experiment, a total of 5,780 and 1,893 peaks were identified in the positive and negative ion modes, respectively. After quality control, 1,093 metabolites were obtained in the positive ion mode and 325 metabolites were obtained in the negative ion mode. The PCA model showed that the cumulative interpretation rates, explaining the statistical variation in X, R2X(cum), were 0.857 in positive ion mode and 0.654 in negative ion mode. The quality control samples gathered together in positive and negative ion modes (Additional file 3: Figure S1).

### Difference in metabolites between 6 and 12 M

**Fig. 1:**
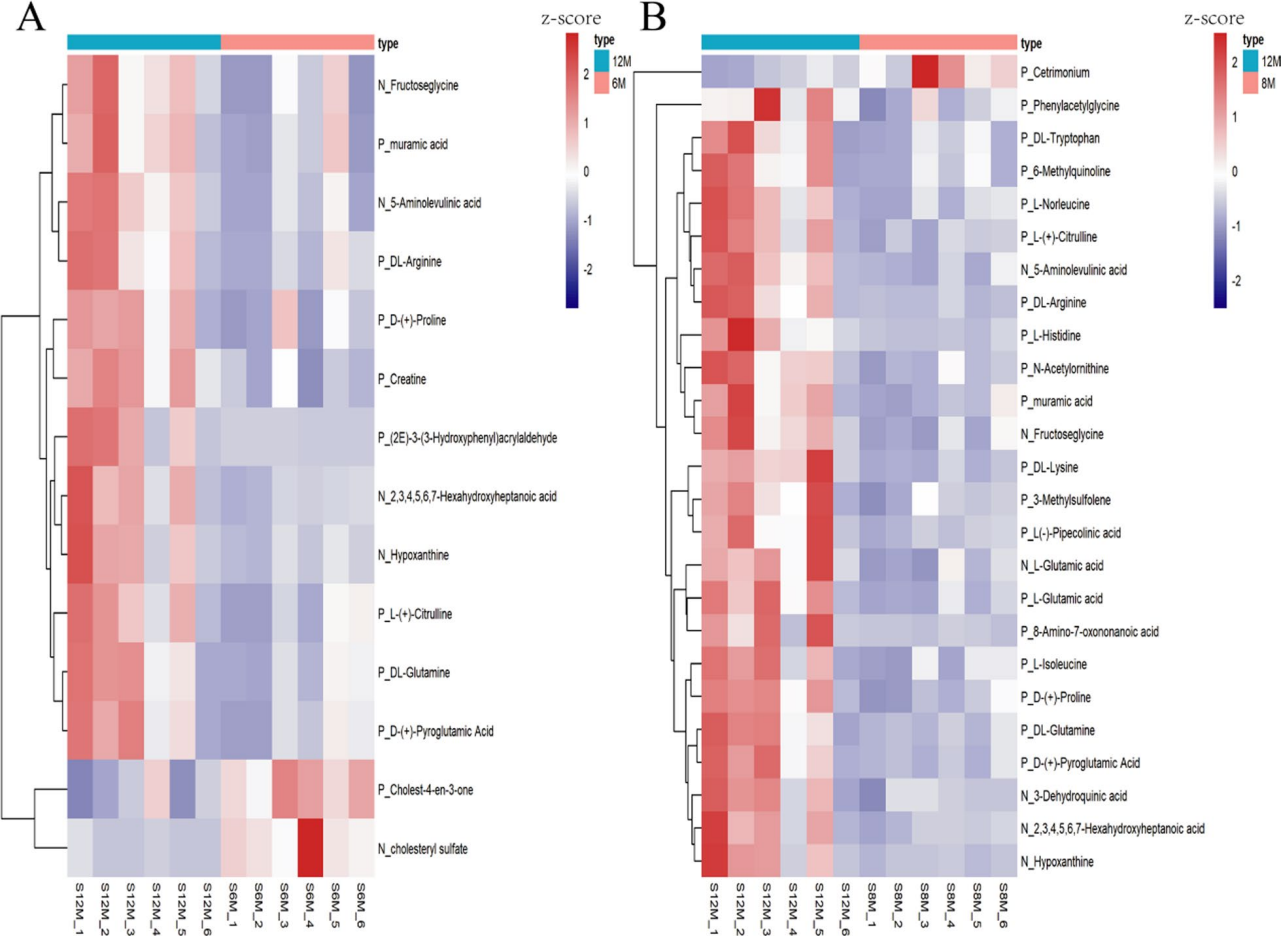
The heatmap of different metabolites. **a** The heatmap of different metabolites between 6 and 12 M, **b** the heatmap of different metabolites between 8 and 12 M

**Fig. 2:**
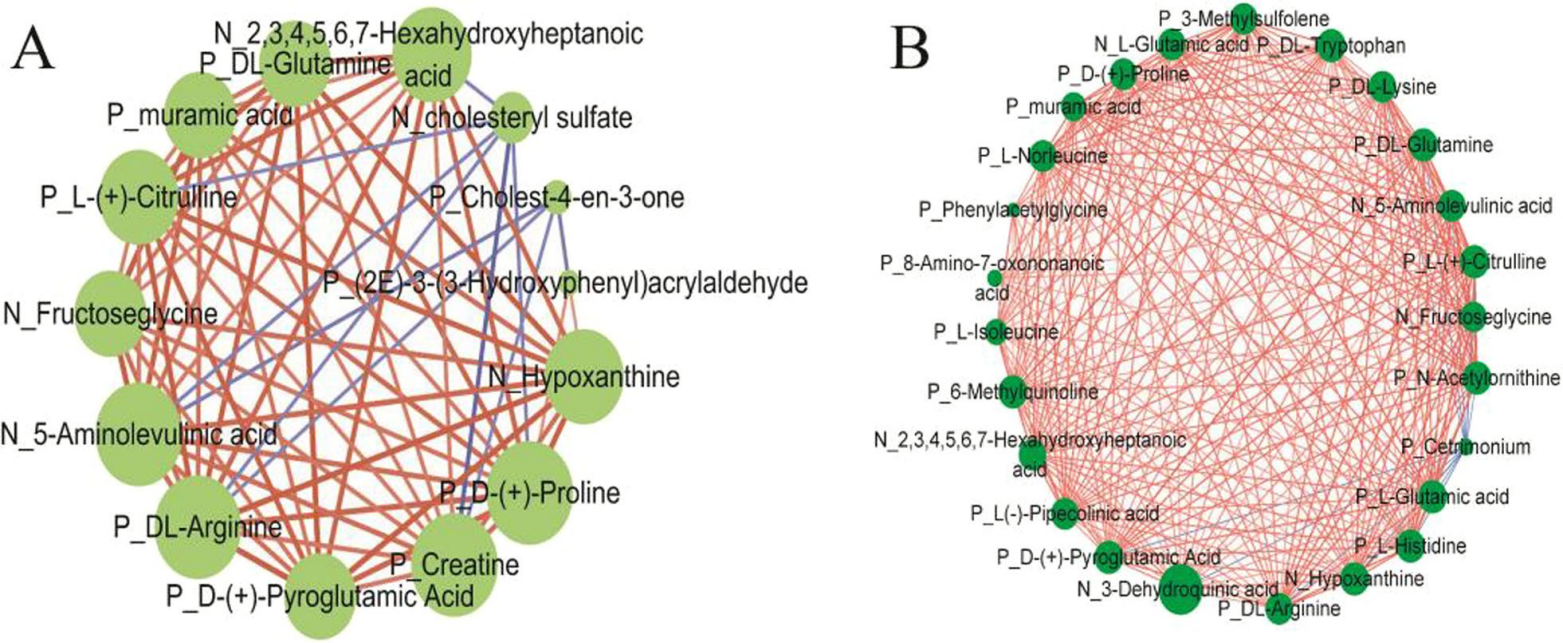
The co-occurrence network of different metabolites. **a** The co-occurrence network between 6 and 12 M, **b** the co-occurrence network between 8 and 12 M. The circle shapes were the different metabolites. The red lines mean the significant positive correlation; the blue lines mean the significant negative correlation. The size of lines mean the correlation size; the size of shapes mean the numbers of networks; the P was positive ion model and the N was negative ion model

The OPLS-DA showed that the samples from 6 and 12 M were well separated. The statistical variation in Y (R2Y) in positive and negative ion modes was above 0.99, and the forecast ability based on the model (Q2) in positive and negative ion modes was 0.370 and 0.692, respectively (Additional file 4: Figure S2 A, B). The Q2 was over 0.3 in both ion modes, which means that the modes were stable and reliable. The intercepts of permutation tests in both ion modes were below 0.05, which means that the models were not overfitted (Additional file 4: Figure S2 C, D). The Q2 and intercepts of permutation tests showed that our models can be used in the next analyses. A total of 14 metabolites were identified as differential metabolites between 6 and 12 M (Fig. 1a; Additional file 5: Table S3). The pathway analyses showed that the 14 differential metabolites were enriched in eight metabolic pathways, including arginine and proline metabolism, glycine and serine metabolism, urea cycle, aspartate metabolism, glutathione metabolism, porphyrin metabolism, bile acid biosynthesis and purine metabolism (Additional file 6: Figure S3). According to the co-occurrence networks, a total of 67 significance relationship pairs were obtained (*P* < 0.05), including the d-(+)-proline, 5-aminolevulinic acid, dl-arginine, creatine, 2,3,4,5,6,7-hexahydroxyheptanoic acid, l-(+)-citrulline, hypoxanthine, fructoseglycine, dl-glutamine, muramic acid, and d-(+)-pyroglutamic acid. Of those, metabolites that formed significant relationship pairs with over 70% of the differential metabolites, were determined as the core significant differential metabolites in 6 M vs. 12 M-old pigs (Fig. 2a).

### Difference in metabolites between 8 and 12 M

The samples from 8 and 12 M were also well separated in OPLS-DA. The R2Ys in positive and negative ion modes were over 0.99, and the Q2s in positive and negative ion modes were 0.414 and 0.708, respectively (Additional file 7: Figure S4A, B). The intercepts of the permutation tests in both ion modes were less than -0.107 (Additional file 7: Figure S4 C, D). Similar to 6 M vs. 12 M, the Q2 and intercepts of permutation tests in 8 M vs. 12 M showed that our model can be used in the next analysis. A total of 25 metabolites were identified as differential metabolites between 6 and 12 M (Fig. 1b) (Additional file 8: Table S4). The pathway analyses showed that the 25 differential metabolites were enriched in 29 metabolic pathways, like arginine and proline metabolism, urea cycle, lysine degradation, and other pathways (Additional file 9: Figure S5). According to the co-occurrence networks, a total of 260 significance relationship pairs were obtained (*P* < 0.05), including 6-methylquinoline, dl-tryptophan, dl-glutamine, N-acetylornithine, l-(+)-citrulline, d-(+)-pyroglutamic acid, l-histidine, hypoxanthine, l-glutamic acid, 2,3,4,5,6,7-hexahydroxyheptanoic acid, 3-methylsulfolene, d-(+)-proline, l-norleucine, 5-aminolevulinic acid, l(−)-pipecolinic acid, dl-arginine, dl-lysine, l-glutamic acid, 3-dehydroquinic acid, fructoseglycine, muramic acid, and l-isoleucine. Of these, metabolites with significant relationships with over 70% of the identified differential metabolites, were determined as the core significant differential metabolites in 8 M vs. 12 M-old pigs (Fig. 2b).

### Analyzing the trends in different age groups

The abundant of significant differential metabolites in the comparisons of 6 M vs. 12 M and 8 M vs. 12 M were used to complete the trend analysis. It resulted in two clusters that were significantly enriched (cluster 21 and cluster 29). The level of cluster 29 increased with age, including dl-tryptophan, phenylacetylglycine, muramic acid, 6-methylquinoline, N-acetylornithine, and l-(−)-pipecolinic acid. In addition, cluster 42 (creatine) and cluster 23 (cholesteryl sulfate) increased and decreased, respectively, with an increase in age (Additional file 10: Figure S6).

### Metabolic profiles in different age groups

**Fig. 3:**
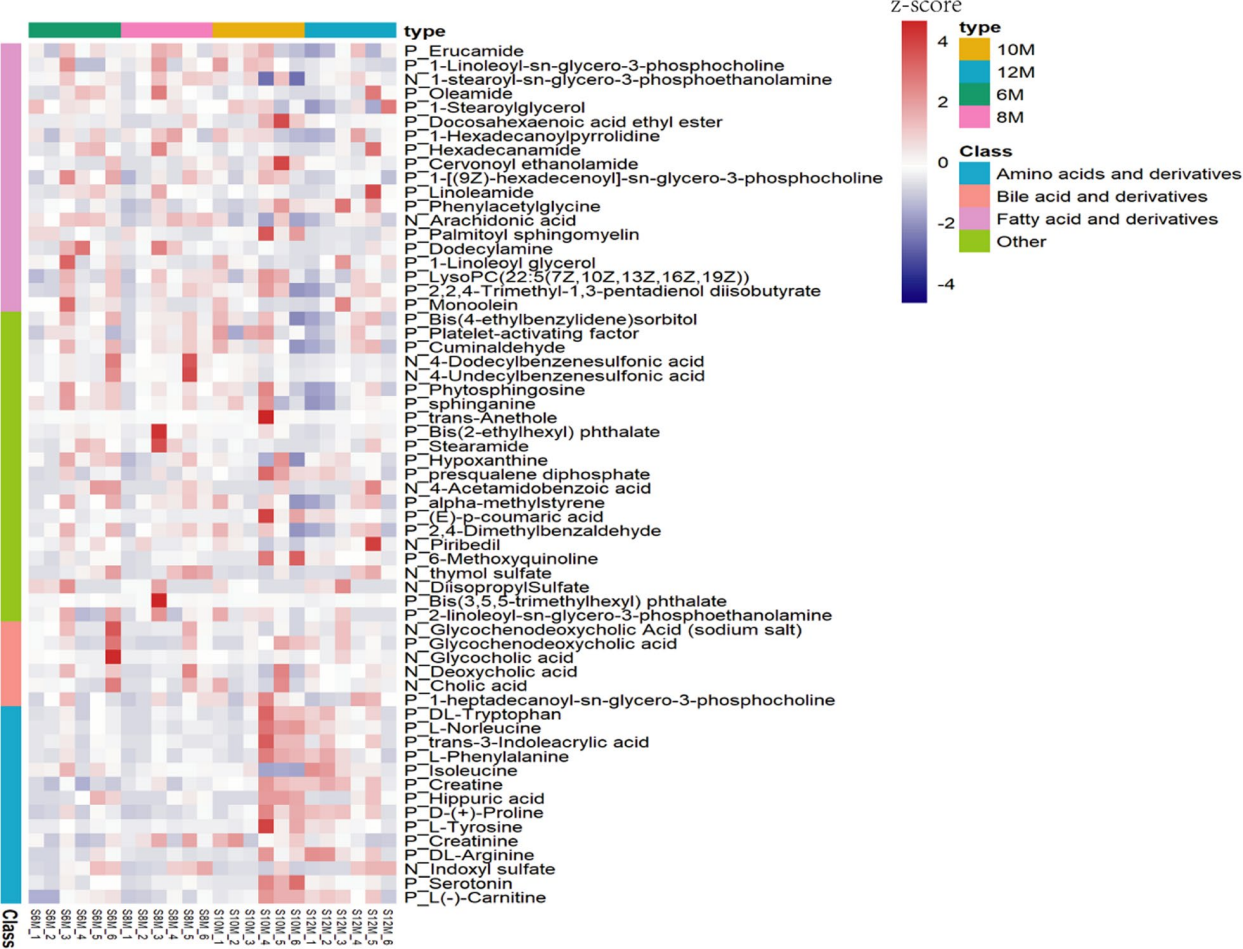
The metabolic profiles in different age groups. The P was positive ion model and the N was negative ion model

A total of 61 out of the top 100 most abundant metabolites was annotated using formula and molecular weight, including 14 amino acids and derivatives (dl-tryptophan, l-norleucine, trans-3-indoleacrylic acid, l-phenylalanine, isoleucine, creatine, hippuric acid, d-(+)-proline, l-tyrosine, creatinine, dl-arginine, indoxyl sulfate, serotonin and l(−)-carnitine), six bile acids and derivatives (glycochenodeoxycholic acid (sodium salt), glycochenodeoxycholic acid, glycocholic acid, deoxycholic acid, cholic acid and 1-heptadecanoyl-sn-glycero-3-phosphocholine), 19 fatty acids and derivatives,(erucamide, 1-linoleoyl-sn-glycero-3-phosphocholine, 1-stearoyl-sn-glycero-3-phosphoethanolamine, oleamide, 1-stearoylglycerol, docosahexaenoic acid ethyl ester, 1-hexadecanoylpyrrolidine, hexadecanamide, cervonoyl ethanolamide, 1-[(9Z)-hexadecenoyl]-sn-glycero-3-phosphocholine, linoleamide, phenylacetylglycine, arachidonic acid, palmitoyl sphingomyelin, dodecylamine, 1-linoleoyl glycerol LysoPC (22:5(7Z,10Z,13Z,16Z,19Z)), 2,2,4-Trimethyl-1,3-pentadienol diisobutyrate, and monoolein) and 22 others (Fig. 3). Amongst the fatty acids and derivatives, only phenylacetylglycine in 8 M-old pigs was significantly lower than of 12 M-old ones. Amongst the amino acids and derivatives, dl-tryptophan, l-norleucine, d-(+)-proline and dl-arginine in 8 M-old pigs was significantly lower than when 12 M old, creatine, d-(+)-proline, while dl-arginine in was significantly lower in 6 M than in 12 M-old pigs.

## Discussion

To explore the blood physiological characteristics, we analyzed 20 blood indices, including seven WBC indices, eight RBC indices, and five platelet indices in 6 M-, 8 M-, 10 M-, and 12 M-old Bama miniature pigs. A total of 13 BPIs showed no significant difference with age, especially all platelet indices. The platelet is formed from megakaryocytes and regulates hemostasis and thrombosis [29]. A low platelet number means acute leukemia [30] and Gaucher's disease [31], usually. Here, however, the platelet indices of healthy Bama miniature pigs could not be related with disease, which might explain why they did not change over time. The lymphocyte ratio, neutrophil ratio, MID cell number, hemoglobin concentration, and hematocrit in different groups varied in Bama miniature pigs between the ages 6 M to 12 M. However, the MID cell ratio increased until the age of 12 M, and the RBC number increased from 8 to 12 M. The MID cell ratio is the percentage of monocytes, eosinophils, and basophils in the blood cell [32]. The monocytes play a key role in immune defense, inflammation, and tissue remodeling [33]. The eosinophils play an important role in immune responses, combating parasites, and bacterial and viral defense [34]. The basophils play a crucial role in regulating innate and acquired immunity, allergic reactions, and autoimmunity [35]. Previous studies report that the RBC numbers in five-week-old pigs was higher than that in weaning pigs [36]. Zhang et al. showed that the RBC number of pigs after five weeks was higher than in the initial stages of growth [37]. Seven BPIs in Bama miniature pigs were closer to the human BPI reference intervals compared with the mouse BPIs, including the neutrophil ratio, lymphocyte number, RBC number, among others. Thus, our study illustrates that the MID cell ratio and RBC number increased with increasing age and the immune function improved gradually from 6 to 12 M in Bama miniature pigs. Generally, the pig as a model organism exhibits many advantages because of their similarity in body size, anatomy, physiology, and immunology to humans, compared to that of mice. Thus, using the pig as a medical model organism may be more suitable to study many kinds of human diseases than the medical model organism mouse. According to the BPIs, the major differences in the metabolic profiles may found between the age groups 6 M and 12 M, and 8 M and 12 M. Therefore, the metabolomics analyses were focused on 6 M-, 8 M-, and 12 M-old pigs.

Non-targeted metabolomics technology was used to examine the content of metabolites in serum samples of Bama miniature pigs. The OPLS-DA analysis indicates significant changes in metabolites because of age. The results of this study suggest that the OPLS-DA models between 6 and 12 M and 8 M and 12 M were reliable, stable, and devoid of overfitting. However, the OPLS-DA model between 10 and 12 M was not reliable, which means the metabolites in the serum in 10 M and 12 M did not differ significantly. These results were consisted with the BPIs in Bama miniature pigs. The relative concentrations of nine amino acids and their derivative metabolites in 6 M-old pigs were significantly lower than when 12 M old. These amino acids were identified as the core significantly differential metabolites: fructoseglycine, dl-glutamine, d-(+)-proline, muramic acid, 5-aminolevulinic acid, l-(+)-citrulline, dl-arginine, d-(+)-pyroglutamic acid, and creatine. Meanwhile, the pathway of four of the eight amino acid metabolism pathways were arginine and proline metabolism, glycine and serine metabolism, aspartate metabolism, and glutathione metabolism pathways. The relative concentrations of 18 metabolites of the amino acids and their derivatives in 8 M-old pigs were significantly lower than those in 12 M-old pigs. They constitute the core significantly differential metabolites: phenylacetylglycine, dl-tryptophan, l-isoleucine, d-(+)-proline, l(−)-pipecolinic acid, dl-glutamine, muramic acid, d-(+)-pyroglutamic acid, l-(+)-citrulline, *N*-acetylornithine, l-norleucine, fructoseglycine, 5-aminolevulinic acid, l-glutamic acid, l-histidine, dl-arginine, and dl-lysine. The 15 identified pathways, out of 29 amino acid metabolism pathways, are: arginine and proline metabolism, lysine degradation, aspartate metabolism, glutathione metabolism, methylhistidine metabolism, glycine and serine metabolism, beta-alanine metabolism, histidine metabolism, alanine metabolism, valine, leucine and isoleucine degradation, cysteine metabolism, phenylalanine and tyrosine metabolism, glutamate metabolism, tryptophan metabolism, and tyrosine metabolism. Zhang et al. showed that leucine, phenylalanine, valine, and isoleucine levels increased with age in the mouse [38]. Peng et al. illustrated that in chicken, the amino acids such as threonine, glutamine, and homoserine on incubation day 19 were substantially higher than on incubation day 14 [39]. In Bama miniature pigs, we see the same negative correlation: the lower the amino acid concentration and their derivatives, the lower is the blood metabolic characteristics in 6 M- and 8 M-old pigs than in 12 M-old pigs, and the amino acid metabolism function may be enhanced with an increase in age. As per the trend analyses, six amino acids and derivatives (dl-tryptophan, phenylacetylglycine, muramic acid, *N*-acetylornithine, l(−)-pipecolinic acid, and creatine) increased in concentration with increasing age. These amino acids are necessary for maintenance, growth, reproduction [40], and immunity, and, therefore, the higher levels of amino acids in 12 M-old pigs may mean further improvement of body function in Bama miniature pigs.

The concentrations of fatty acids and derivatives in 12 M-old pigs were lower than those in 6 M-, 8 M-, and 10 M-old pigs. On the contrary and inversely proportional, the concentrations of amino acids and derivatives in 12 M-old pigs were higher than those in 6 M-, 8 M-, and 10 M-old pigs. The fatty acid concentration increases in obese individuals [41] and the fat deposition is enhanced with age [42]. This opposite trend was unexpected and has not been observed in other studies, yet. Thus, the function of fatty acids and derivatives in 12 M-old pigs and the relationship between fatty acids and amino acids in Bama miniature pigs need further study.

In addition, it is worth remembering that using the BPIs and serum metabolite reference values which report on our study should refer to the guideline of ASVCP, since these parameters are affected by the age, health level, breed and other factors.

## Conclusion

In this study, we analyzed the different BPIs and serum metabolites to identify the changes in blood profiles in Bama miniature pigs between 6 to 12 M of age. Our results show that the MID cell ratio and RBC number increase over time and identify the pig as a more suitable medical model organism than the mouse. The lower the concentration of amino acids and derivatives, the lower is the blood metabolic characteristics in 6 M- and 8 M-old Bama miniature pigs than in 12 M-old pigs. The metabolic profiles contained 14 amino acids and derivatives, six bile acids and derivatives, 19 fatty acids and derivatives, and 22 others, and the concentration of fatty acids and derivatives are inversely proportional to amino acids and derivatives.

## Supplementary Information

**Additional file 1. Table S1.** Instrument operation program of UPLC-MS/MS analysis.

**Additional file 2. Table S2.** Changes of solvents in gradient elution of UPLC-MS/MS analysis.

**Additional file 3. Figure S1.** The Principal component analysis (PCA) score plot.

**Additional file 4. Figure S2.** The orthogonal partial least squares discriminant analysis (OPLS-DA) analysis between 6M and 12M.

**Additional file 5. Table S3.** Information of 14 different metabolites in positive ion between 6M and 12M.

**Additional file 6. Figure S3**. The enriched KEGG between 6M and 12M.

**Additional file 8. Figure S4.** The orthogonal partial least squares discriminant analysis (OPLS-DA) analysis between 8M and 12M.

**Additional file 8. Table S4.** Information of 25 different metabolites in positive ion between 8M and 12M.

**Additional file 9. Figure S5.** The enriched KEGG between 8M and 12M.

**Additional file 10. Figure S6.** The different metabolites cluster.

## Data Availability

The complete dataset for this study can be accessed at 10.6084/m9.figshare.20238051.v1.

## Abbreviations

6 M: Six month old
8 M: Eight month old
10 M: Ten month old
12 M: Twenty month old
BPIs: Blood physiological indexes
WBC: White blood cells
RBC: Red blood cells
UPLC-MS: Ultraperformance LC–MS
MS: Mass spectrum
PCA: Unsupervised principal component analysis
OPLS-DA: Supervised orthogonal partial least squares discriminant analysis
VIP: Variable importance in projection
FC: Fold changes
P: P-value
KEGG: Encyclopedia of Genes and Genomes in the Kyoto Protocol
HMDB: Human metabolome database
STEM: Short time-series expression miner
R2X: The interpretation of variation in X
R2Y: The interpretation of variation in Y
QC: Quality control
Q2: Forecast ability based on the model
