# Supplementary material for: Blood metabolic and physiological profiles of Bama miniature pigs at different growth stages
Source: Porcine Health Manag. 2022 Aug 8;8:35. doi: 10.1186/s40813-022-00278-7 (PMC9358802; doi:10.1186/s40813-022-00278-7)
Supplement: Supplementary file 1 — Additional file 1. Table S1. Instrument operation program of UPLC-MS/MS analysis. [file 40813_2022_278_MOESM1_ESM.doc]

Table S1 Instrument operation program of UPLC-MS/MS analysis

| Team | Parameter | Item | Parameter |
| --- | --- | --- | --- |
| Column temperature | 30 ℃ | Mass range | 80 m/z to 1200 m/z |
| Autosampler temperature | 4 ℃ | Sheath gas flow rate | 30 psi |
| Sample injection volume | 2 µl | Auxiliary gas flow rate | 10 psi |
| Mobile phase A | Water plus 0.1% formic acid | Transmission Capillary temperature | 320 ℃ |
| Mobile phase B | Methanol | Primary scan resolutions | 70 000 |
| Spray voltage | 3.0 kV | Secondary scan resolutions | 17500 |
