# Supplementary material for: Blood metabolic and physiological profiles of Bama miniature pigs at different growth stages
Source: Porcine Health Manag. 2022 Aug 8;8:35. doi: 10.1186/s40813-022-00278-7 (PMC9358802; doi:10.1186/s40813-022-00278-7)
Supplement: Supplementary file 2 — Additional file 2. Table S2. Changes of solvents in gradient elution of UPLC-MS/MS analysis. [file 40813_2022_278_MOESM2_ESM.doc]

Table S2 Changes of solvents in gradient elution of UPLC-MS/MS analysis

| Time /min | Mobile phase A(%) | Mobile phase B(%) |
| --- | --- | --- |
| 0-2 | 95-80 | 5-20 |
| 2-12 | 80-5 | 20-95 |
| 12-17 | 5-5 | 95-95 |
| 17-17.1 | 5-95 | 95-5 |
| 17.1-20 | 95-95 | 5-5 |
