# Supplementary material for: Blood metabolic and physiological profiles of Bama miniature pigs at different growth stages
Source: Porcine Health Manag. 2022 Aug 8;8:35. doi: 10.1186/s40813-022-00278-7 (PMC9358802; doi:10.1186/s40813-022-00278-7)
Supplement: Supplementary file 3 — Additional file 3. Figure S1. The Principal component analysis (PCA) score plot. [file 40813_2022_278_MOESM3_ESM.doc]

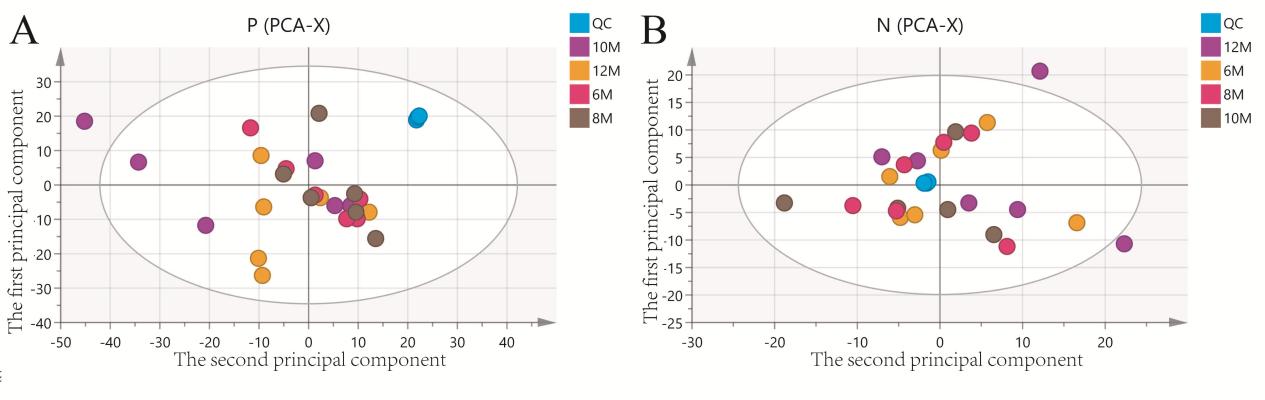


Figure S1 The Principal component analysis (PCA) score plot. (A) PCA score plot for the three groups analyzed in the positive ion mode. (B) PCA score plot for the three groups analyzed in the negative ion mode.
