# Supplementary material for: Blood metabolic and physiological profiles of Bama miniature pigs at different growth stages
Source: Porcine Health Manag. 2022 Aug 8;8:35. doi: 10.1186/s40813-022-00278-7 (PMC9358802; doi:10.1186/s40813-022-00278-7)
Supplement: Supplementary file 4 — Additional file 4. Figure S2. The orthogonal partial least squares discriminant analysis (OPLS-DA) analysis between 6M and 12M. [file 40813_2022_278_MOESM4_ESM.doc]

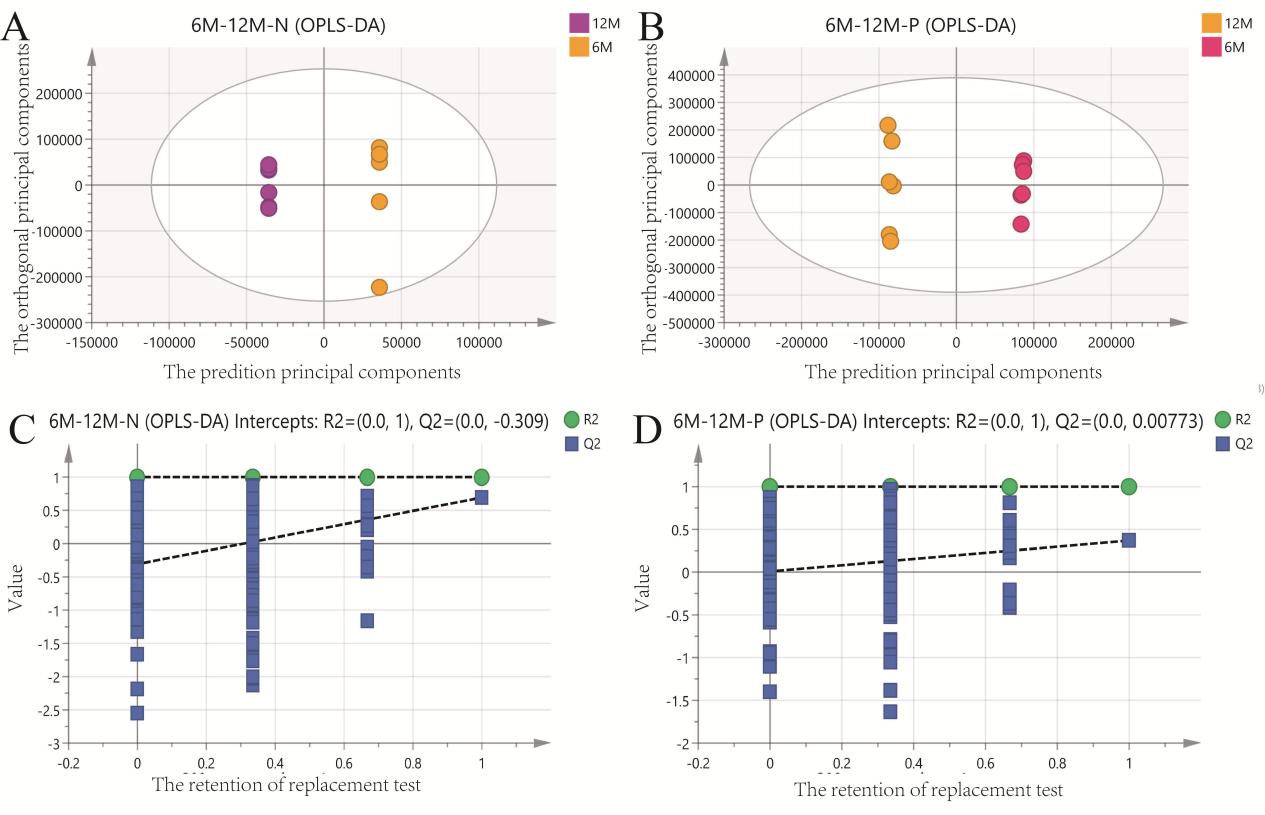


Figure S2 The orthogonal partial least squares discriminant analysis (OPLS-DA) analysis between 6M and 12M. A: The OPLS-DA score plot for the two groups analyzed in the negative ion mode, B: The OPLS-DA score plot for the two groups analyzed in the positive ion mode. C: The OPLS-DA permutation test plot for the two groups analyzed in the negative ion mode. D: The OPLS-DA permutation test plot for the two groups analyzed in the positive ion mode.
