# Supplementary material for: Blood metabolic and physiological profiles of Bama miniature pigs at different growth stages
Source: Porcine Health Manag. 2022 Aug 8;8:35. doi: 10.1186/s40813-022-00278-7 (PMC9358802; doi:10.1186/s40813-022-00278-7)
Supplement: Supplementary file 5 — Additional file 5. Table S3. Information of 14 different metabolites in positive ion between 6M and 12M. [file 40813_2022_278_MOESM5_ESM.doc]

Table S3 Information of 14 different metabolites in positive ion between 6M and 12M

| Name | Formula | Molecular Weight (Da) | VIP value | Fold Change (6M/12M) | HMDB number |
| --- | --- | --- | --- | --- | --- |
| 2,3,4,5,6,7-Hexahydroxyheptanoic acid | C7 H14 O8 | 226.07 | 2.22 | 0.20 | HMDB0240292 |
| Fructoseglycine | C8 H15 N O7 | 237.08 | 1.11 | 0.35 | HMDB0060278 |
| (2E)-3-(3-Hydroxyphenyl)acrylaldehyde | C9 H8 O2 | 148.05 | 5.81 | 0.08 | HMDB0040986 |
| Cholest-4-en-3-one | C27 H44 O3 | 416.33 | 1.09 | 2.71 | HMDB0012459 |
| DL-Glutamine | C5 H10 N2 O3 | 146.07 | 2.15 | 0.40 | HMDB0003423 |
| D-(+)-Proline | C5 H9 N O2 | 115.06 | 2.96 | 0.50 | HMDB0003411 |
| Muramic acid | C9 H17 N O7 | 251.10 | 1.53 | 0.33 | HMDB0003254 |
| 5-Aminolevulinic acid | C5 H9 N O3 | 131.06 | 1.34 | 0.24 | HMDB0001149 |
| L-(+)-Citrulline | C6 H13 N3 O3 | 175.10 | 1.10 | 0.37 | HMDB0000904 |
| Cholesteryl sulfate | C27 H46 O4 S | 466.31 | 1.08 | 11.98 | HMDB0000653 |
| DL-Arginine | C6 H14 N4 O2 | 174.11 | 3.45 | 0.31 | HMDB0000517 |
| D-(+)-Pyroglutamic Acid | C5 H7 N O3 | 129.04 | 1.75 | 0.43 | HMDB0000267 |
| Hypoxanthine | C5 H4 N4 O | 136.04 | 1.68 | 0.19 | HMDB0000157 |
| Creatine | C4 H9 N3 O2 | 131.07 | 5.27 | 0.37 | HMDB0000064 |
