# Supplementary figures and images for: Blood metabolic and physiological profiles of Bama miniature pigs at different growth stages
Source: Porcine Health Manag. 2022 Aug 8;8:35. doi: 10.1186/s40813-022-00278-7 (PMC9358802; doi:10.1186/s40813-022-00278-7)

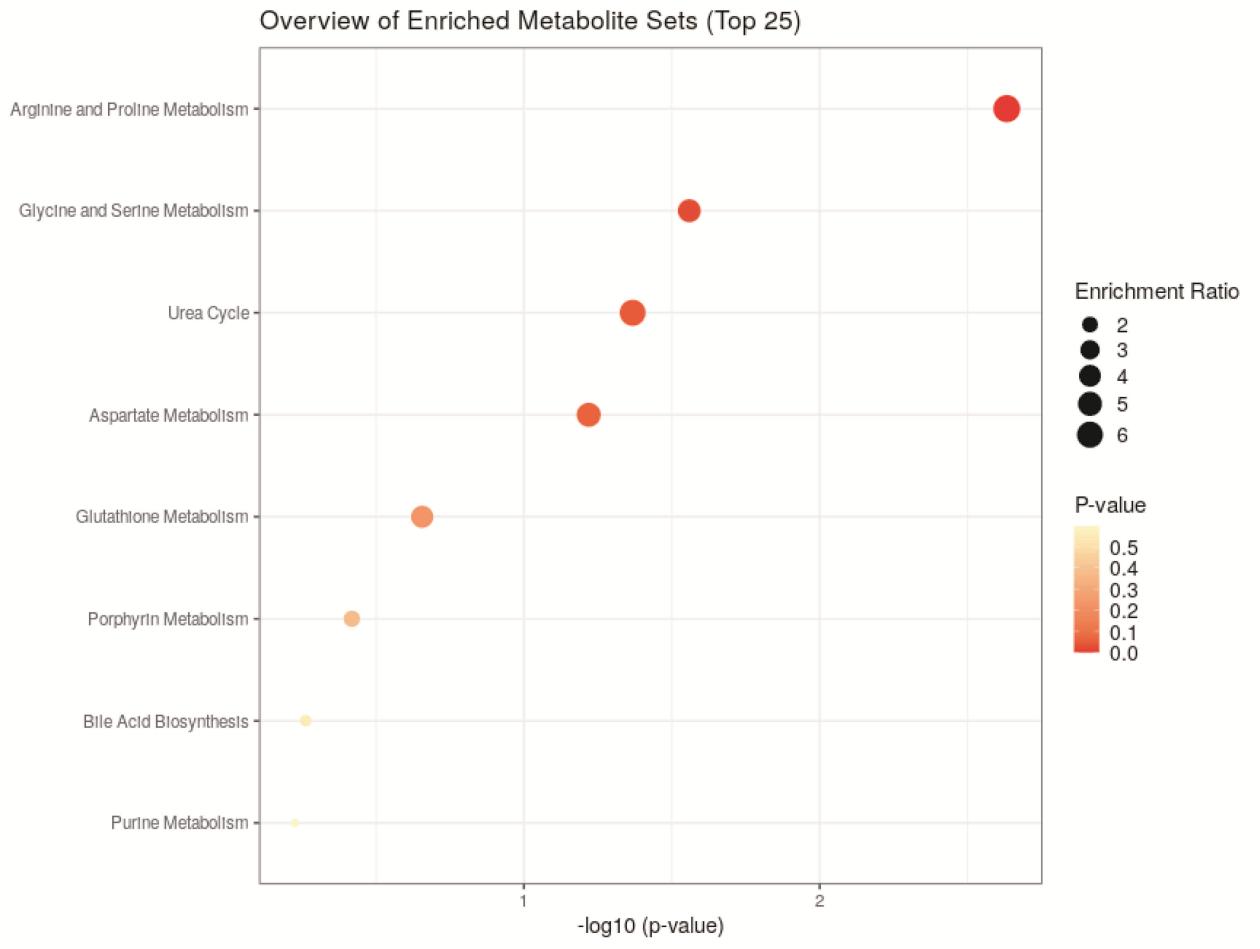


Figure S3 The enriched KEGG between 6M and 12M.

Supplement: Supplementary file 6 — Additional file 6. Figure S3. The enriched KEGG between 6M and 12M. [file 40813_2022_278_MOESM6_ESM.doc]

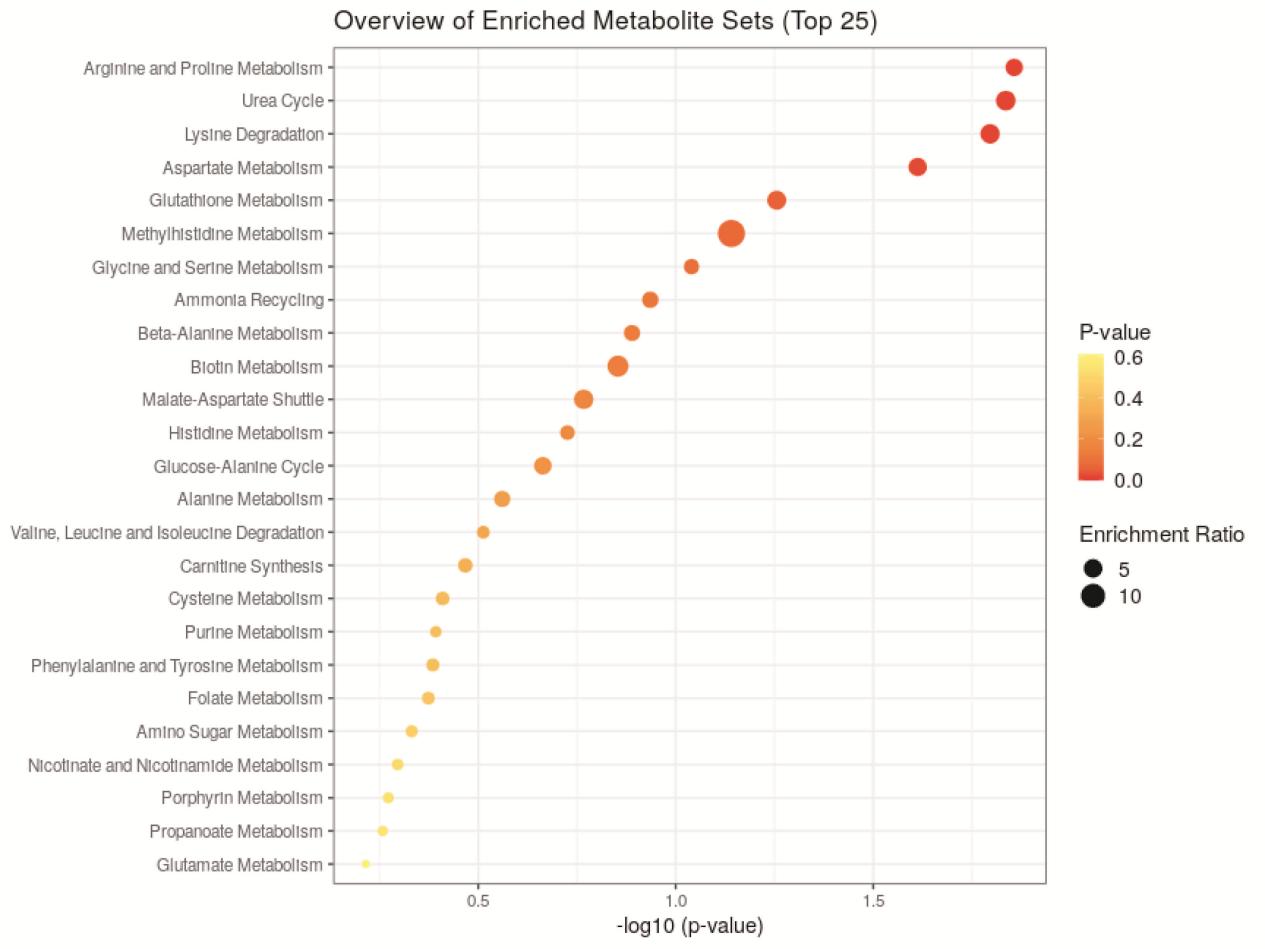


Figure S5 The enriched KEGG between 8M and 12M.

Supplement: Supplementary file 9 — Additional file 9. Figure S5. The enriched KEGG between 8M and 12M. [file 40813_2022_278_MOESM9_ESM.doc]

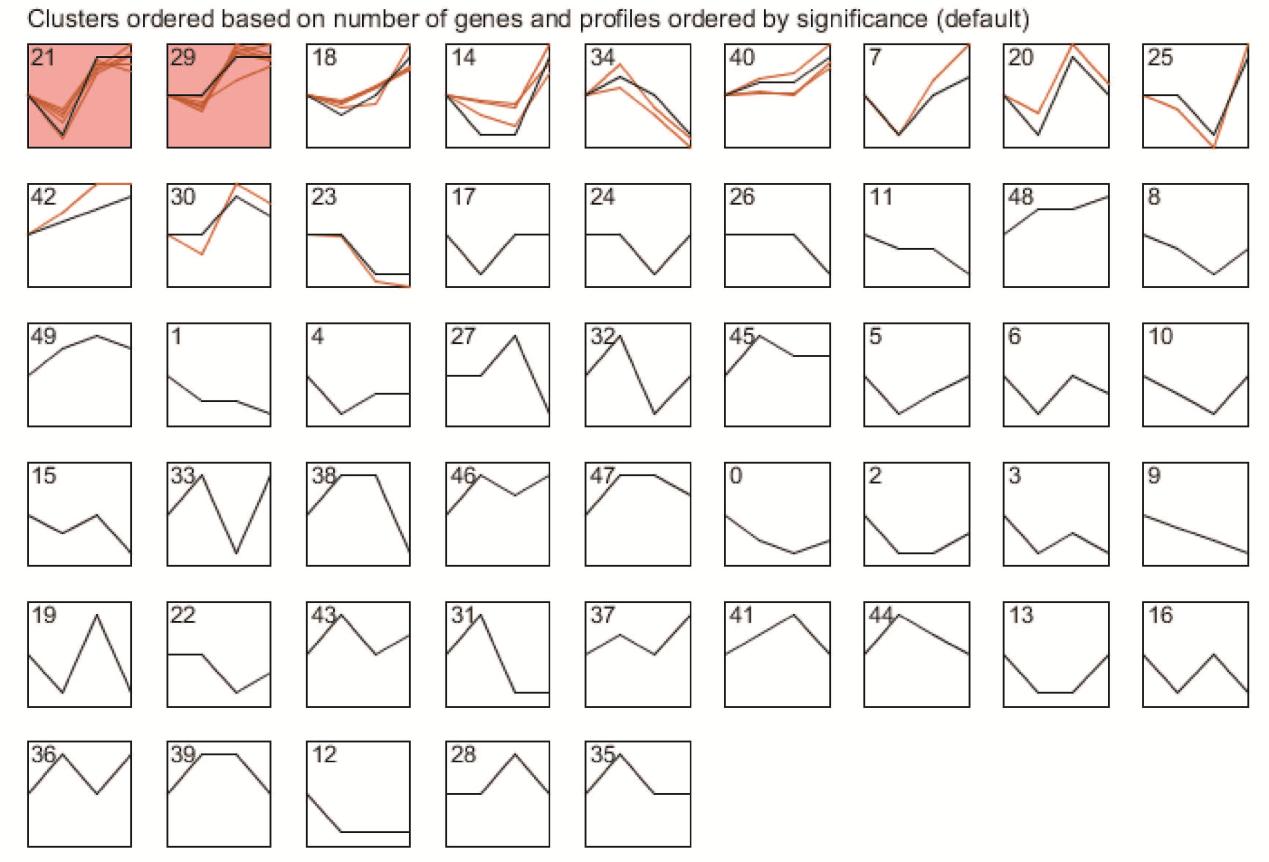


Figure S6 The different metabolites cluster.

Supplement: Supplementary file 10 — Additional file 10. Figure S6. The different metabolites cluster. [file 40813_2022_278_MOESM10_ESM.doc]
