# Supplementary material for: Blood metabolic and physiological profiles of Bama miniature pigs at different growth stages
Source: Porcine Health Manag. 2022 Aug 8;8:35. doi: 10.1186/s40813-022-00278-7 (PMC9358802; doi:10.1186/s40813-022-00278-7)
Supplement: Supplementary file 8 — Additional file 8. Table S4. Information of 25 different metabolites in positive ion between 8M and 12M. [file 40813_2022_278_MOESM8_ESM.doc]

Table S4 Information of 25 different metabolites in positive ion between 8M and 12M

| Name | Formula | Molecular Weight (Da) | VIP value | Fold Change (8M/12M) | HMDB number |
| --- | --- | --- | --- | --- | --- |
| Cetrimonium | C19 H41 N | 283.32 | 1.46 | 4.08 | - |
| 3-Methylsulfolene | C5 H8 O2 S | 132.02 | 1.11 | 0.49 | HMDB0059667 |
| Phenylacetylglycine | C10 H11 N O3 | 193.07 | 2.57 | 0.47 | HMDB0000821 |
| 3-Dehydroquinic acid | C7 H10 O6 | 190.05 | 1.05 | 0.39 | HMDB0012710 |
| DL-Tryptophan | C11 H12 N2 O2 | 204.09 | 7.16 | 0.38 | HMDB0013609 |
| L-Isoleucine | C6 H13 N O2 | 131.09 | 3.44 | 0.37 | HMDB0000172 |
| 6-Methylquinoline | C10 H9 N | 143.07 | 1.07 | 0.37 | HMDB0033115 |
| D-(+)-Proline | C5 H9 N O2 | 115.06 | 3.26 | 0.37 | HMDB0003411 |
| L(-)-Pipecolinic acid | C6 H11 N O2 | 129.08 | 1.24 | 0.34 | HMDB0000716 |
| DL-Glutamine | C5 H10 N2 O3 | 146.07 | 2.11 | 0.33 | HMDB0003423 |
| Muramic acid | C9 H17 N O7 | 251.10 | 1.41 | 0.33 | HMDB0003254 |
| D-(+)-Pyroglutamic Acid | C5 H7 N O3 | 129.04 | 1.82 | 0.32 | HMDB0000267 |
| L-(+)-Citrulline | C6 H13 N3 O3 | 175.10 | 1.06 | 0.32 | HMDB0000904 |
| N-Acetylornithine | C7 H14 N2 O3 | 174.10 | 1.20 | 0.31 | HMDB0003357 |
| L-Norleucine | C6 H13 N O2 | 131.09 | 6.85 | 0.31 | HMDB0001645 |
| Fructoseglycine | C8 H15 N O7 | 237.08 | 1.15 | 0.30 | HMDB0060278 |
| 5-Aminolevulinic acid | C5 H9 N O3 | 131.06 | 1.25 | 0.27 | HMDB0001149 |
| L-Glutamic acid | C5 H9 N O4 | 147.05 | 1.19 | 0.27 | HMDB0000148 |
| 2,3,4,5,6,7-Hexahydroxyheptanoic acid | C7 H14 O8 | 226.07 | 2.02 | 0.24 | HMDB0240292 |
| L-Glutamic acid | C5 H9 N O4 | 147.05 | 1.62 | 0.23 | HMDB0000148 |
| L-Histidine | C6 H9 N3 O2 | 155.07 | 1.29 | 0.22 | HMDB0000177 |
| DL-Arginine | C6 H14 N4 O2 | 174.11 | 3.45 | 0.21 | HMDB0000517 |
| Hypoxanthine | C5 H4 N4 O | 136.04 | 1.64 | 0.16 | HMDB0000157 |
| DL-Lysine | C6 H14 N2 O2 | 146.11 | 1.31 | 0.16 | HMDB0000182 |
| 8-Amino-7-oxononanoic acid | C9 H17 N O3 | 187.12 | 1.61 | 0.12 | HMDB0240687 |
